# Thanks to the Editorial Board of Dementia & Neuropsychology

**DOI:** 10.1590/1980-5764-DN-2025-A001

**Published:** 2025-03-21

The Editors of Dementia & Neuropsychology thank the Co-Editors (2), Social Media and Visual Abstract Editor (1), Associate Editors (50), Consultive Board Members (50), and Referees (144), who have altruistically and without payment dedicated their time to carrying out the editorial tasks of this journal from January 1, 2024, to December 31, 2024.

Thank you very much!

## CO-EDITORS

Eliane Correa Miotto

Mônica Sanches Yassuda

## SOCIAL MEDIA AND VISUAL ABSTRACT EDITORS

Ricardo Alvim

## ASSOCIATE EDITORS

Ceres Eloah de Lucena Ferretti

Claudia Berlim de Mello

Claudia Kimie Suemoto

Cláudia Maia Memória

Cleusa Pinheiro Ferri

Déborah Oliveira

Douglas Mendes Nunes

Eliasz Engelhardt

Elisa de Paula França Resende

Ezequiel Surace

Florindo Stella

Jacy Bezerra Parmera

Jerusa Smid

Joaquim Brasil Neto

Juliana Nery de Souza-Talarico

Karolina Gouveia César Freitas

Lea Tenenholz Grinberg

Leandro Tavares Lucato

Leonardo Cruz de Souza

Leonel Tadao Takada

Lucas Porcello Schilling

Maíra Okada de Oliveira

Maira Tonidandel Barbosa

Marcela Lima Silagi de Siqueira

Marcia Cristina Nascimento Dourado

Marcia Maria Pires Camargo Novelli

Marcio Luiz Figueredo Balthazar

Maria Joana Mader Joaquim

Maria Niures Pimentel dos Santos Matioli

Mario Amore Cecchini

Mauricio Arcos-Burgos

Mirna Lie Hosogi Senaha

Nilton Santos Custodio Capuñay

Pablo Miguel Bagnati

Raphael Machado de Castilhos

Renata Kochhann

Roberta Diehl-Rodriguez

Rubens Gisbert Cury

Thaís Bento Lima da Silva

Valéria Santoro Bahia

Valeska Marinho Rodrigues

Vitor Geraldi Haase

Vitor Tumas

Wyllians Jose Vendramini Borelli

## CONSULTIVE BOARD

Alexandre Castro-Caldas

André Luis Fernandes Palmini

Andrea Camaz Deslandes

Andrew J. Lees

Benito Pereira Damasceno

Cláudia da Costa Leite

Claudia Sellitto Porto

Dora Selma Fix Ventura

Facundo F. Manes

Francisco de Assis Carvalho do Vale

Francisco Javier Lopera Restrepo (In Memoriam)

Giacomo Rizzolatti

Helenice Charchat-Fichman

Henrique Cerqueira Guimarães

Howard Chertkow

Jamary Oliveira Filho

Jerson Laks

João Carlos Barbosa Machado

John C. Morris

John R. Hodges

Jorge Neval Moll Neto

José Luiz Sá Calvalcanti

Kenichi Meguro

Leila Maria Cardao Chimelli

Leonardo Ferreira Caixeta

M.-Marsel Mesulam

Márcia Lorena Fagundes Chaves

Marcia Radanovic

Maria Alice de Mattos Pimenta Parente

Maria Rita dos Santos e Passos Bueno

Mayana Zatz

Michael D. Geschwind

Moyses de Paula Rodrigues Chaves

Orestes Vicente Forlenza

Patricio Fuentes

Paulo Caramelli

Paulo Henrique Ferreira Bertolucci

Paulo Roberto de Brito Marques

Renato Anghinah

Ricardo de Oliveira Souza

Ricardo F. Allegri

Sandra Weintraub

Sérgio Luís Blay

Sergio Teixeira Ferreira

Tales Alexandre Aversi-Ferreira

Tânia Marcourakis

Thomas H. Bak

Vilma Regina Martins

Wilson Jacob Filho

Yves Joanette

## REVIEWERS

Álvaro de Oliveira Franco

Álvaro Pentagna

Ana Claudia Vieira

Ana Luiza Navas

Ana Luiza Nunes Cunha

Ana Paula Bertholo

Ana Tubero

Analuiza Camozzato

Ananda Falcone

André Palmini

Andrei Bieger

Andrew Miguel

Angélica Dal Pizzol

Antonio Alvim

Antonio Lucio Teixeira

Artur Coutinho

Augusto Penalva de Oliveira

Benito Damasceno

Breno Barbosa

Bruno Iepsen

Cammy Leung

Carlos Pomilio

Ceres Ferretti

Christopher H. Schmid

Claudia Memória

Claudia Drummond

Claudia Mello

Clineu Almada

Conrado Borges

Cristiano Aguzzoli

Daiane Prudêncio

Daniela Durand

Danusa Machado

Deborah Gonzalez

Diego Chambergo-Michilot

Eliane Miotto

Eliasz Engelhardt

Elisa de Paula França Resende

Felipe de Oliveira

Flavia Garcez

Florindo Stella

Giancarlo Lucchetti

Helio Teive

Henrique Ballalai-Ferraz

Henrique Salmazo da Silva

Henrique Silva

Howard Ribeiro Junior

Isabela Thaís Machado de Jesus

Isabela Vernaglia

Isabelle Patriciá F. Soares Chariglione

Ivan Moya Diez

Jason Soble

Jerusa Smid

Jessica Plácido

Jonas de Paula

Juliana Bonini

Juliana Lira

Juliana Souza-Talarico

Juliana Tonaki

Karen Socher

Karin Ortiz

Karina Pagliarin

Karine Porpino Viana

Kette Valente

Lambros Messinis

Lara Araujo

Leila Maria Chimelli

Leonel Takada

Letícia Sampaio

Liana Fernandez

Lucas Mourão

Luciana Fonseca

Luciana Passuello

Luís Felipe Miranda

Luis Leitão

Luís Sousa

Luziany Araujo

Madson Alan Maximiano-Barreto

Maicon Albuquerque

Maira Okada de Oliveira

Maira Olchik

Marcela Silagi de Siqueira

Marcelo de Brito

Marcelo Gaete Fernandez

Márcia Cominetti

Marcio Luiz Balthazar

Marcos Castello de Oliveira

Mari Nilva Maia da Silva

Maria Niures Matioli

Maria Paula Foss

Maria Rita Gascón

Mario Melo

Mauricio Teixeira

Maysa Cera

Millene Camilo

Monica Santoro Yassuda

Natalia Gonçalves

Neelufar Shama Shaik

Nilton Custodio Capuñay

Norberto Frota

Patricia Aparecida Zuanetti

Patrícia Freitas

Paula Nunes

Paulo Bertolucci

Paulo Caramelli

Pedro Barbosa

Rafael Carra

Rahman Tanveer

Raphael Castilhos

Raphael Spera

Raquel Costa

Renata Kochhann

Renato Luiz Marchetti

Ricardo Alvim

Ricardo Nitrini

Rizaldy Taslim Pinzon

Rodrigo Nicolato

Rogerio Beato

Roy Rillera Marzo

Rui Rothe-Neves

Satiko Andrezza Ferreira

Sebastian Walsh

Sheila Trentin

Silvia Merlin

Simone Barreto

Stewart Longman

Susanny Vercellino Tassini

Taiza Stumpp

Takano

Tamine Capato

Tatiana Santos

Tatiane da Silva

Thais Bento Lima da Silva

Thamires Magalhães

Thiago Cordeiro

Tomas Leon

Verônica Araujo

Victor Calil

Victor de Carvalho Brito Pontes

Vilma Regina Martins

Vinay Vakharia

Vitor Ribeiro Paes

Vitor Tumas

Wellington Oliveira

Wyllians Borelli

